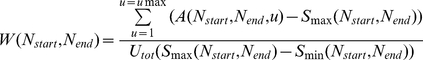# Gradients and Modulation of K^+^ Channels Optimize Temporal Accuracy in Networks of Auditory Neurons

**DOI:** 10.1371/journal.pcbi.1002424

**Published:** 2012-03-15

**Authors:** Leonard K. Kaczmarek

**Affiliations:** 1 Department of Pharmacology, Yale University School of Medicine, New Haven, Connecticut, United States of America; 2 Department of Cellular and Molecular Physiology, Yale University School of Medicine, New Haven, Connecticut, United States of America; Université Paris Descartes, Centre National de la Recherche Scientifique, France

## Abstract

Accurate timing of action potentials is required for neurons in auditory brainstem nuclei to encode the frequency and phase of incoming sound stimuli. Many such neurons express “high threshold” Kv3-family channels that are required for firing at high rates (>∼200 Hz). Kv3 channels are expressed in gradients along the medial-lateral tonotopic axis of the nuclei. Numerical simulations of auditory brainstem neurons were used to calculate the input-output relations of ensembles of 1–50 neurons, stimulated at rates between 100–1500 Hz. Individual neurons with different levels of potassium currents differ in their ability to follow specific rates of stimulation but all perform poorly when the stimulus rate is greater than the maximal firing rate of the neurons. The temporal accuracy of the combined synaptic output of an ensemble is, however, enhanced by the presence of gradients in Kv3 channel levels over that measured when neurons express uniform levels of channels. Surprisingly, at high rates of stimulation, temporal accuracy is also enhanced by the occurrence of random spontaneous activity, such as is normally observed in the absence of sound stimulation. For any pattern of stimulation, however, greatest accuracy is observed when, in the presence of spontaneous activity, the levels of potassium conductance in all of the neurons is adjusted to that found in the subset of neurons that respond better than their neighbors. This optimization of response by adjusting the K^+^ conductance occurs for stimulus patterns containing either single and or multiple frequencies in the phase-locking range. The findings suggest that gradients of channel expression are required for normal auditory processing and that changes in levels of potassium currents across the nuclei, by mechanisms such as protein phosphorylation and rapid changes in channel synthesis, adapt the nuclei to the ongoing auditory environment.

## Author Summary

In order to detect the nature and location of a sound stimulus, neurons in the central auditory system have to fire at very high rates with extreme temporal precision. Specifically, they have to be able to follow changes in an auditory stimulus at rates of up to 2000 Hz or more and to lock their action potentials to the stimuli with a precision of only a few microseconds. An individual neuron, however, cannot fire at such high rates, and the intrinsic electrical properties of neurons, such as the relative refractory period that follows each action potential, severely limits accuracy of timing at high rates. The intrinsic excitability of neurons is governed by the potassium channels that they express. It has been found in auditory brainstem nuclei that there exist gradients of these channels such that each neuron typically has a different number of channels than its neighbors. In this study, computational models based on measurements in auditory neurons demonstrate that, in the presence of random spontaneous activity such as is normally observed in auditory neurons, rapid adjustments of levels of potassium current within neurons along the gradient are required to allow the ensemble to transmit accurate timing information. The findings suggest that regulation of potassium channels within gradients is an integral component of auditory processing.

## Introduction

Transfer of information in the auditory system occurs at rates that are faster than can be accomplished by single neurons. Accurate responses to auditory stimuli frequently require the discrimination of events that are separated in time by only tens of microseconds, and the ability to follow changes in the stimulus at rates of up to several thousand Hertz. In contrast, neuronal action potentials typically have durations close to a millisecond and the most rapidly firing neurons can generate action potentials at only several hundred Hertz.

Experimental clues on how the central nervous system copes with high rates of information transfer have come from studies of auditory brainstem neurons, such as those of the anteroventral cochlear nucleus (AVCN) and the medial nucleus of the trapezoid body (MNTB) [1], [2], [3], [4], [5]. Among other targets, these neurons project to the lateral and medial superior olives (LSO and MSO), where information that is relayed through the nuclei is used to detect microsecond differences in the timing of auditory stimuli, as well as small differences in their amplitude, to calculate the positions of auditory stimuli in space [1], [6].

Neurons in the AVCN and MNTB lock their action potentials very precisely in time to the phase of sounds at frequencies up to ∼3000 Hz [7], [8], [9], [10], [11] and/or lock their action potentials to the temporal envelope of higher frequency sounds that are amplitude-modulated at frequencies up to ∼3000 Hz [12]. Nevertheless, individual neurons in these nuclei have maximal firing rates of only a few hundred Hz [13], [14], [15]. In order to follow higher rates of auditory signals, these phase-locking neurons respond selectively to only a subset of the stimuli. For example, in response to a 600 Hz auditory stimulus, a neuron that fires at a maximal rate of 350 Hz may lock its action potentials to every other cycle of the stimulus, effectively firing at only 300 Hz.

The limitations of the intrinsic excitability of neurons raise some fundamental questions. For example, how can a neuron differentiate between two patterns of synaptic inputs, one at 600 and the other at 1200 Hz if it fires at exactly 300 Hz in response to each of these stimuli? How can small differences in frequency be detected in the face of the high degree of spontaneous activity that occurs even in the absence of sound stimulation? In the MNTB, the phase-locking principal neurons fire *in vivo* at rates between 10 to 300 Hz even in silence [7], [10]. This spontaneous activity is believed to be generated by spontaneous transmitter release from sensory hair cells in the cochlea [16], [17], [18], [19].

The resolution of some of these questions is that a sound stimulus in encoded in the activity of an ensemble of neurons rather than by single neurons. The accuracy of the output of an ensemble is, however, dependent on the number and characteristics of its individual neurons. A variety of modeling studies, as well as experimental studies, have documented that the intrinsic electrical properties of neurons in nuclei such as the AVCN and MNTB play a key role in determining their responses to synaptic inputs triggered by auditory stimuli [20], [21], [22], [23], [24], [25]. These intrinsic electrical properties are, however, not uniform but vary across the lateral-to-medial tonotopic axis of these nuclei. For example, levels of the voltage-dependent “high-threshold” K^+^ channel Kv3.1 are low in the lateral, low-frequency part of the MNTB and high in the medial, high-frequency aspect of the nucleus [26], [27], [28], [29], [30], [31]. In contrast, some other “lower-threshold” channels such as, Kv1.3 [32], Na^+^-activated K^+^ channels [33], and an intermediate voltage-activated K^+^ current [26] are expressed in an opposite gradient with highest levels in lateral MNTB neurons.

This manuscript describes numerical simulations of the firing patterns of ensembles of neurons with firing properties based on those of phase-locking auditory brainstem neurons. It is demonstrated that, in the absence of spontaneous activity, the accuracy of timing of outputs is enhanced in ensembles that have gradients of high-threshold K^+^ channels over those that have uniform levels of these channels. Paradoxically, accuracy of phase-locking is also substantially enhanced by random spontaneous activity in the input to the neurons, even when the neurons have uniform electrical properties. For any stimulus pattern in the presence of spontaneous activity, greatest temporal accuracy is obtained when levels of the high-threshold K^+^ current in all neurons are adjusted to a specific uniform level. This specific level corresponds to the level found in those neurons in the gradient that initially respond to the stimulus pattern more accurately than their neighbors.

The present simulations provide a potential biological explanation for the existence of gradients of ion channels across neurons of a single nucleus. They also suggest that the relatively rapid modulation of K^+^ channels within gradients reflect an adaptation that allows the auditory system to adjust the processing of timing information to different auditory environments. Modulation of K^+^ channels has been found to occur in response to changes in ongoing auditory activity [30], [34], and is mediated by mechanisms such as channel phosphorylation [34], [35], [36] and synthesis of new channel proteins [29], [37], [38].

## Results

### Temporal accuracy depends on levels of K^+^ current

**Figure 1:**
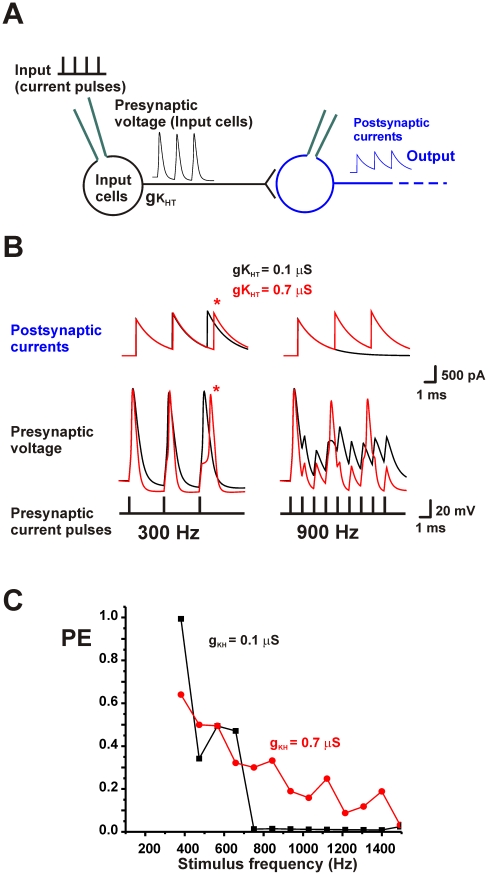
Intrinsic excitability of neurons introduces timing errors. A. Diagram of elements used in the numerical simulations. B. Computed traces for a single model neuron with a value of gK_HT_ of 0.1 or 0.7 µS showing presynaptic voltage waveforms and postsynaptic currents at the onset of a 300 Hz or 900 Hz stimulus train. Errors in timing are apparent at 300 Hz with gK_HT_ = 0.7 µS (asterisk). At 900 Hz, the neuron with gK_HT_ = 0.1 µS fires only a single action potential at the onset of the train. C. A plot of the vector strength PE for this neuron with gK_HT_ equal to 0.1 or 0.7 µS as a function of stimulus frequency.

To examine the accuracy with which phase-locking neurons are capable of transmitting timing information, simulations of the pattern of firing of model neurons with ionic currents based on those recorded in MNTB neurons in brain slices were first carried out [39]. The models that describe the patterns of firing of the presynaptic neurons used in this study have been described in detail previously [34], [36], [40], [41], [42]. Individual model neurons (Input cells) were stimulated by brief current pulses (250 µs) at rates of >100 Hz (Input, Fig. 1A). When the upstroke of action potentials crossed 0 mV, axonal propagation that triggered the presynaptic release of neurotransmitter was assumed to occur. A second trace, representing the postsynaptic effects of the stimulus train, was then calculated. Postsynaptic currents, with a decay time constant of 2 msec, were triggered by each presynaptic action potential (Output, Fig. 1A). For convenience, calculations were made for excitatory postsynaptic currents, such as those evoked in AVCN neurons by inputs from the auditory nerve, or in MNTB neurons from the AVCN input. Exactly the same general principles would apply, however, to inhibitory postsynaptic currents, such as those evoked in superior olivary neurons by inputs from the MNTB.

To calculate the timing accuracy of the postsynaptic responses with respect to the stimulus train, the strength of a phase vector that evaluates how accurately the evoked postsynaptic responses are locked to individual stimuli during a 100 msec train was calculated. For each postsynaptic trace the height and time of the maximal peak in current that occurred between one stimulus pulse and the next was determined. The strength of the phase vector was then calculated as described previously [34], [41]. The strength of this phase vector was then multiplied by a factor that reflects the proportion of stimuli that evoked a postsynaptic response (see Methods) [29]. For a neuron with one postsynaptic output this factor is the same as entrainment, i.e. it reflects the proportion of input stimuli that triggered action potentials in the input cells. This adjusted parameter was termed PE (for Phase/Entrainment). For example if only 50% of stimuli evoke responses, the maximal value of PE is 0.5.

At lower stimulus rates, the neurons are capable of firing an action potential in response to each stimulus. This is seen in the left panels of Fig. 1B, which shows the presynaptic action potentials and postsynaptic currents at the onset of a 300 Hz stimulus train. If each action potential and postsynaptic response has the same latency with respect to its stimulus pulse the strength of the vector PE can come close to the theoretical maximum value of one. The exact timing of the action potentials, however, depends on the level of high-threshold Kv3 current (K_HT_) in the presynaptic neuron. As has been found previously in pharmacological, genetic knockout and modeling studies, changes in K_HT_ current produce only minor changes in action potential width but have a major impact on the response to repetitive stimulation [34], [36]. Fig. 1B compares the responses for a low conductance (gK_HT_ = 0.1 µS) and a high conductance (gK_HT_ = 0.7 µS) of K_HT_ in the presynaptic neuron. The low K_HT_ conductance produces action potentials that have a uniform delay for each stimulus pulse. In contrast, the timing of the action potentials in the neuron with high K_HT_ is progressively delayed, such that the latency from the onset of the stimulus pulse to the postsynaptic response is clearly different for the third stimulus compared to the first. Such delays have been termed “errors” in timing [34] and are caused by the increased relatively refractory period that results from high levels of voltage-dependent K^+^ conductance.

A different situation occurs when the input neurons are stimulated at higher rates (900 Hz in Fig. 1B). As is also found in pharmacological and genetic knockout studies [36], neurons with low K_HT_ are incapable of generating more than a single action potential at the onset of the train. Conversely, higher levels of K_HT_ allow a neuron to maintain firing throughout the train of rapid input stimuli. This is because the stronger repolarization with high K_HT_ levels allows sodium channels to recover from inactivation more rapidly, and thus maintains firing during the synaptic barrage. Fig. 1C displays plots of the vector strength PE as a function of stimulus frequency for these two different levels of K_HT_.

**Figure 2:**
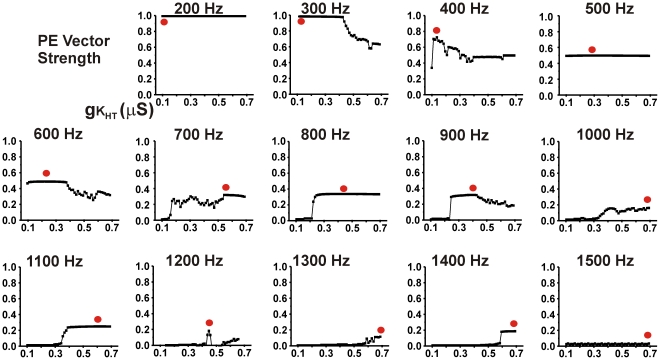
Plots of PE against gK_HT_, the conductance of high threshold Kv3.1-like K_HT_ current at rates of stimulation from 200–1500 Hz. Except for the value of gK_HT_, the properties of the presynaptic neuron are identical to those in Fig. 1. Red dots indicate the values of gK_HT_ that provide the highest value for PE across the range of gK_HT_ from 0.1 to 0.7 µS.

Thus high levels of K_HT_ allow neurons to fire at high rates, but introduce timing errors such as those seen at 300 Hz in Fig. 1A, while neurons with low K_HT_ levels preserve timing accuracy but are incapable of firing at high rates [34], [36]. Fig. 2 shows plots of PE against levels of K_HT_ between 0.1 and 0.7 µS for stimulation rates of 200–1500 Hz. At each stimulus rate, a red circle indicates the level of K_HT_ that provides the optimal PE vector strength for that rate of stimulation. At low rates of stimulation (200–400 Hz) the ability of neurons to follow the stimulus with high temporal accuracy is maximal if the neuron has a low level of K_HT_. As stated above, this is because the relative refractory period after an action potential, which delays the timing of a subsequent action potential, is minimized at low levels of K_HT_ [34]. In contrast, at high rates of stimulation (1000–1500 Hz), neurons with low levels of K_HT_ are incapable of following the stimulus, and high levels of K_HT_ improve the value of PE. At high rates of stimulation, however, individual neurons are incapable of responding to every stimulus, and the maximal value of PE is very low compared to that achieved with low stimulus rates. For intermediate rates of stimulation, optimal PE vector strength occurs in neurons that have intermediate levels of K_HT_.

### Gradients of ion channels improve temporal accuracy of a neuronal ensemble

**Figure 3:**
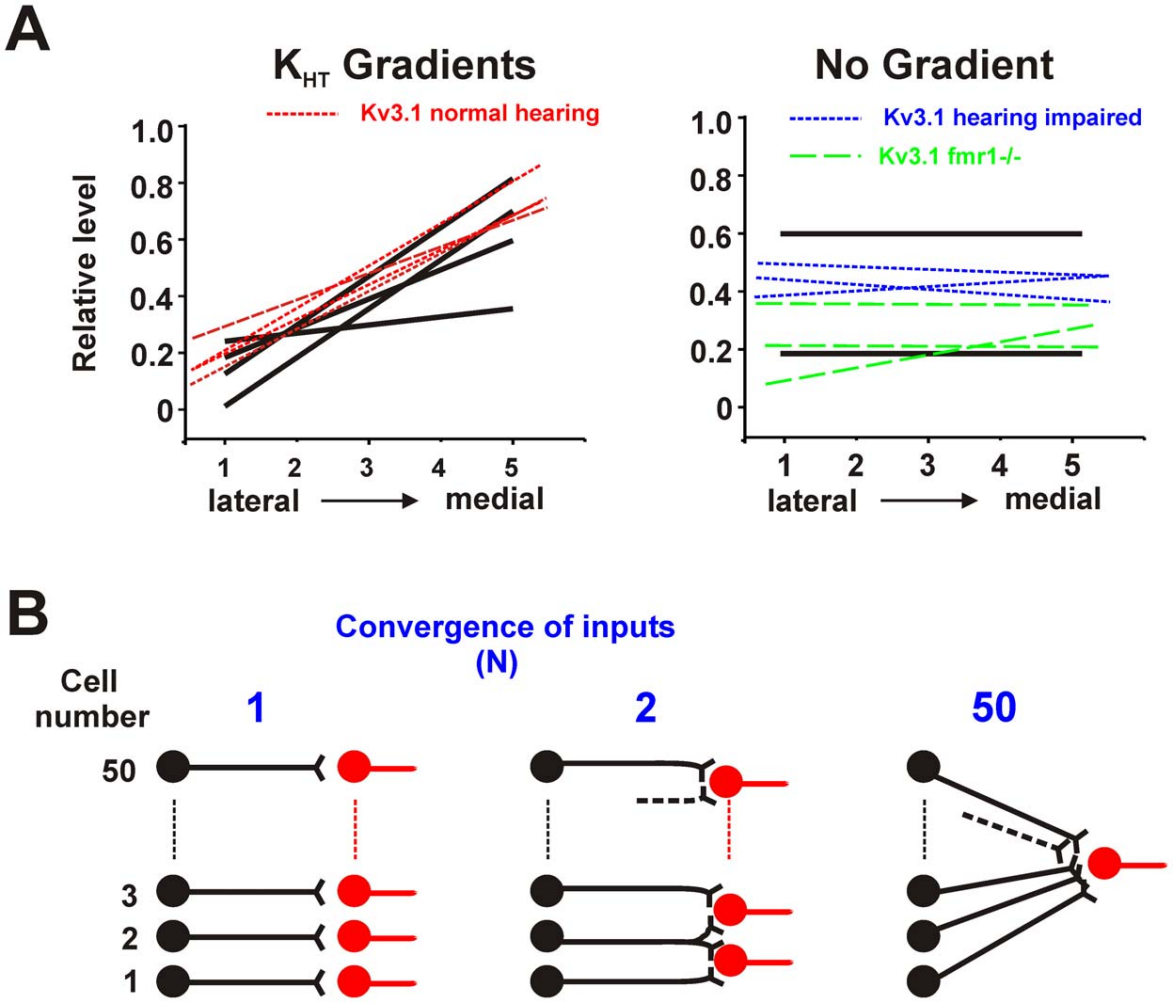
A. Comparison of gradients of the Kv3.1 channel measured along the lateral-medial aspect of the MNTB with gradients of the K_HT_ current used in the present simulations. Left panel shows gradients measured in normal hearing mice (dashed lines) [31] and those used in the simulations in Fig. 4 (solid lines). Right panel shows lack of gradients, or impaired gradients, in hearing-impaired mice (short dash lines) [31], *Fmr1^−/−^* mice (long dash lines) [29] and relative Kv3.1 levels used for no-gradient simulations (solid lines). B. Schematic diagram illustrating convergence of the synaptic outputs of a linear array of 50 neurons. In the left panel each neuron connects only to a single postsynaptic cell, resulting in 50 individual values of PE. In the center panel, pairs of neighboring neurons in the array converge on a single postsynaptic cell (resulting in 49 values of PE). On the right, all neurons in the ensemble converge onto one postsynaptic neuron, resulting in a single unique value for PE.

In animals with normal hearing, levels of high threshold Kv3 channel proteins in auditory brainstem nuclei are not uniform. They are low in the lateral, low-frequency part of nuclei such as the MNTB and AVCN, and progressively increase towards the medial, high-frequency aspect of these nuclei [26], [27], [28], [29], [30], [31]. Fig. 3A shows how the relative levels of Kv3.1 change along this axis of the MNTB in published measurements made on normal-hearing rodents and compares these to data from studies in which Kv3.1 levels become uniform across the nucleus, either because of the onset of partial hearing loss [31] or in an animal model of Fragile-X Syndrome [29]. It also shows the relative gradients of K_HT_ that were used in simulations.

To test the effects of such gradients on temporal accuracy, an ensemble of 50 neurons in which the levels of K_HT_ vary systematically across the ensemble was simulated. For each ensemble, the PE vector strength was first calculated for the postsynaptic output of each individual neuron, and then again calculated for linear combinations of the postsynaptic output of groups of 2, 3, 4, …50 neighboring neurons (Fig. 3B).

An important aspect of the interpretation of the parameter PE for the output of an ensemble is that it no longer reflects the amount of entrainment of individual input neurons by the stimulus input. For example for a stimulus that is greater than the maximal firing rate of a single neuron, the value of PE can still be equal to 1 if the combined postsynaptic outputs of the ensemble are of equal amplitude and delay for every stimulus pulse that is applied, even if each individual neuron fires only in response to a fraction of the input stimulus pulses.

**Figure 4:**
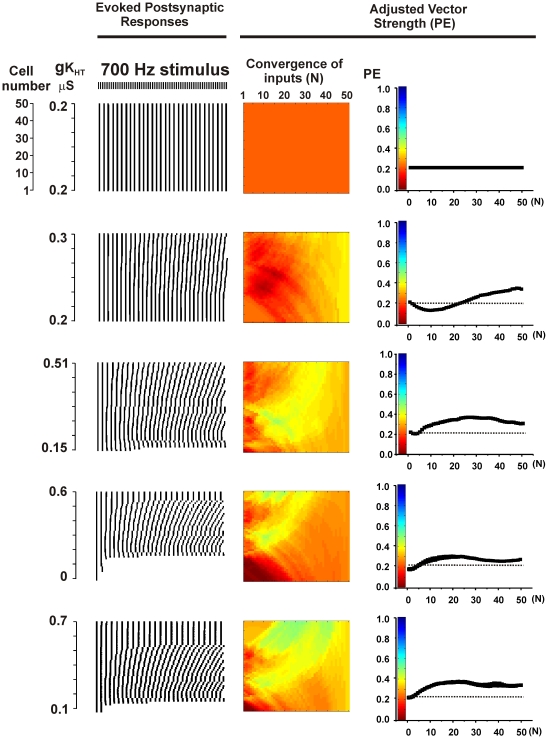
Gradients of K_HT_ channels enhance vector strength PE of combined postsynaptic output. Left panels show raster plots for the timing of the peaks of postsynaptic output of 50 individual neurons. Each peak that follows a stimulus pulse is represented as a short vertical line and these are aligned vertically for the 50 neurons in a linear ensemble. Center panels show a color-coded representation of the vector strength PE for linear combinations of the output from 1 to 50 of the neurons in the ensemble. Colors at the very left of the rectangle represent the value of PE for the outputs of the 50 individual neurons corresponding to the left panels. The color at the right edge of the rectangle represents PE for the combined postsynaptic output of all 50 neurons. Colors in the middle represent the values of PE for linear combinations of 2–49 neurons. Color code is shown in the right panels, which also plot the mean values of PE for linear convergence of the output of 1–50 neighboring presynaptic neurons. Top panels show results for an ensemble with no channel gradient, while the lower four sets of panels show results for the four gradients depicted in Fig. 3A (left panel).

To portray the effect of combining the outputs of multiple neurons from the ensemble of 50 cells, a color code was used to represent the strength of the adjusted phase vector (PE) in which brown corresponds to random firing (V = 0) and dark blue to perfect phase-locking and entrainment in the output (PE = 1) (Fig. 4). The left edge of the rectangle represents 50 points corresponding to PE for the output of individual neurons. The right edge is a single color that represents the unique value of PE when the output of all 50 neurons is combined equally to generate a single postsynaptic trace (as in Fig. 3B *right*). Intermediate points along the x-axis of the rectangle represent the values of PE of linear combinations of 2–49 neighboring neurons along the gradient. The top set of panels of Fig. 4 show the result obtained for an ensemble with a uniform distribution of Kv3.1, stimulated at 700 Hz. If the intrinsic electrical properties of neurons in an ensemble are all identical, and all of the neurons receive the same stimulus, then, as in this case, the combined output of the ensemble is identical to that for a single neuron.

The lower set of panels in Fig. 4 shows results for ensembles with the four gradients depicted in Fig. 2C. Also shown graphically are the mean values of PE for the combined outputs of 1–50 neurons (*right* panels). It is evident that combining the outputs of multiple neurons in a gradient can slightly improve the value of PE over that produced by an ensemble of uniform neurons. This is because the intrinsic properties of the neurons vary across the ensemble and the timing of action potentials triggered by the stimulus train is different for each neuron, as illustrated in the raster plots at the left of Fig. 4. In the combined postsynaptic output, there will be a summation of postsynaptic currents that are triggered by action potentials locked to individual stimulus pulses. Postsynaptic currents resulting from errors in action potential timing can be averaged out in the combined output. Thus the combined output of the ensemble can be enhanced in accuracy over the response of any one cell.

### Spontaneous activity improves temporal accuracy of a neuronal ensemble

**Figure 5:**
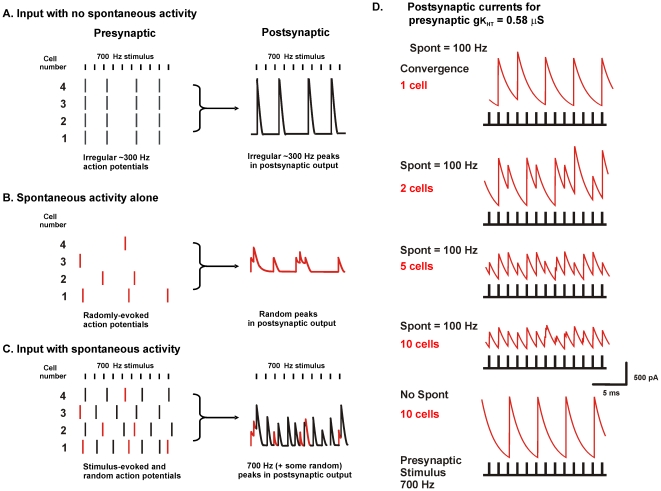
Random stimulation of an ensemble of neurons improves fidelity of timing. A. Schematic diagram illustrating four identical neurons stimulated at 700 Hz. The 700 Hz stimulus results in errors of timing and failures of action potential generation that are propagated to the combined postsynaptic output. B. Random stimulation of the four neurons produces only random output in the combined postsynaptic output. C. If random stimulation is combined with 700 Hz stimulation, the responses of individual neurons to the 700 Hz stimulus are shifted in time with respect to their neighbors. As a result, errors and omissions are averaged out in the combined postsynaptic output, and the ensemble generates a more faithful representation of the 700 Hz stimulus pattern. D. Computed postsynaptic currents for ensembles of 1, 2, 5 or 10 model neurons with a value of K_HT_ = 0.58 µS, during stimulation at 700 Hz in the presence or absence of random spontaneous activity with a mean frequency (Spont) of 100 Hz.

There is a high degree of spontaneous activity in the auditory nerve and in neurons of auditory brainstem nuclei even in the absence of sounds. This spontaneous discharge rate typically varies from ∼10 Hz to over 200 Hz and is believed to be triggered by spontaneous transmitter release from cochlear inner hair cells [7], [10], [16], [17]. As is illustrated schematically in Fig. 5A–C, the occurrence of random spontaneous activity might be expected to shift the pattern of response to an ongoing stimulus. For example, the occurrence of a spontaneous action potential immediately before an incoming stimulus would be expected to delay the response of the neuron to the next stimulus. This would be expected to improve the value of PE by desynchronizing responses during high rates of stimulation so that cycle-skipping is no longer synchronized across the population. Such spontaneous activity would also be expected to randomize the occurrence of stimulus-evoked errors in timing.

Fig. 5D, which shows the effect of introducing spontaneous activity into ensembles with 1, 2, 5 or 10 input neurons, demonstrates that such improvement does in fact occur. Spontaneous activity was applied to individual presynaptic neurons, which all had the same value of K_HT_ (0.58 µS), as brief current pulses identical to those used for the stimulus train but with a random distribution in time (and with a mean frequency of 100 Hz). In the presence of spontaneous activity, the output from the ensemble of 10 neurons has peaks of similar amplitude and delay in response to each of the stimulus pulses that were applied at 700 Hz. In contrast, with no spontaneous activity, the output of the 10 neuron ensemble is identical to that of a single neuron with a K_HT_ value of 0.58 µS. In this case the timing of peaks in the postsynaptic output is limited by the firing rate of the individual neurons.

**Figure 6:**
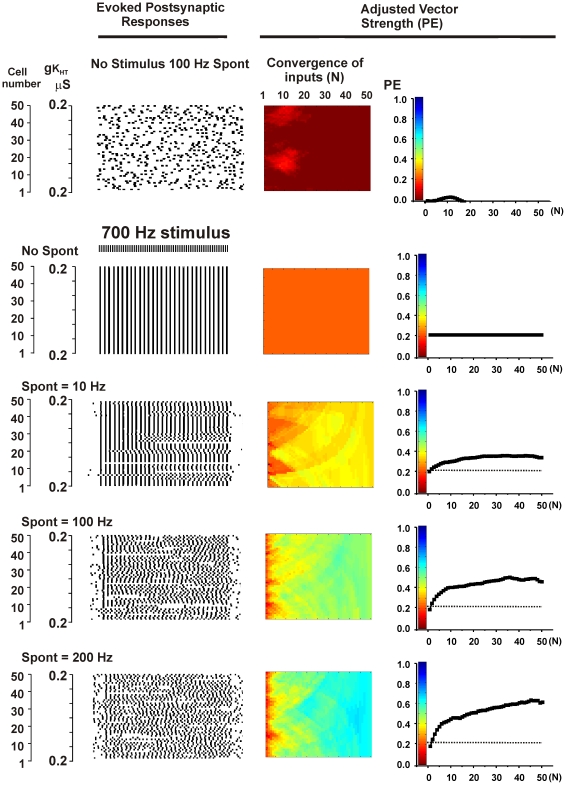
Random spontaneous activity enhances vector strength PE in ensembles of neurons stimulated at high rates. Descriptions of panels are as for Fig. 4. A. Top panels show random spontaneous activity in the absence of any patterned stimulation for an ensemble with no gradient of channel expression (same parameters as top panels in Fig. 4). Values of PE for this condition were calculated with respect to a hypothetical 700 Hz stimulus. The second set of panels show simulations for a 700 Hz stimulus applied to the ensemble with no random spontaneous activity. The bottom three sets of panels show the effect of combining 700 Hz stimulation with random spontaneous activity with a mean frequency (Spont) of 10, 100 or 200 Hz.

A more complete test of the effects of random spontaneous activity was carried out using the ensemble of 50 neurons with uniform electrical properties first shown at the top of Fig. 4. As expected, when random patterns of stimulation were presented to the model ensembles of neurons in absence of a stimulus, calculations of vector strength with respect to any stimulus produced values close to zero (Fig. 6, top panels). When random spontaneous activity at mean rates of 10–200 Hz was combined with a coherent 700 Hz stimulus, however, spontaneous activity greatly enhanced the temporal accuracy of the output of the ensemble (Fig. 6, lower panels). Moreover, increases in the overall rate of spontaneous activity systematically increased the overall phase vector strength of the output. When, however, the rate of spontaneous activity was increased up to and past the rate of the stimulus itself, the steepness of the relation between PE and the number of convergent output cells (N) was progressively decreased, reflecting degradation of the signal (data not shown).

### Changes in K_HT_ in the presence of spontaneous activity enhance temporal accuracy

**Figure 7:**
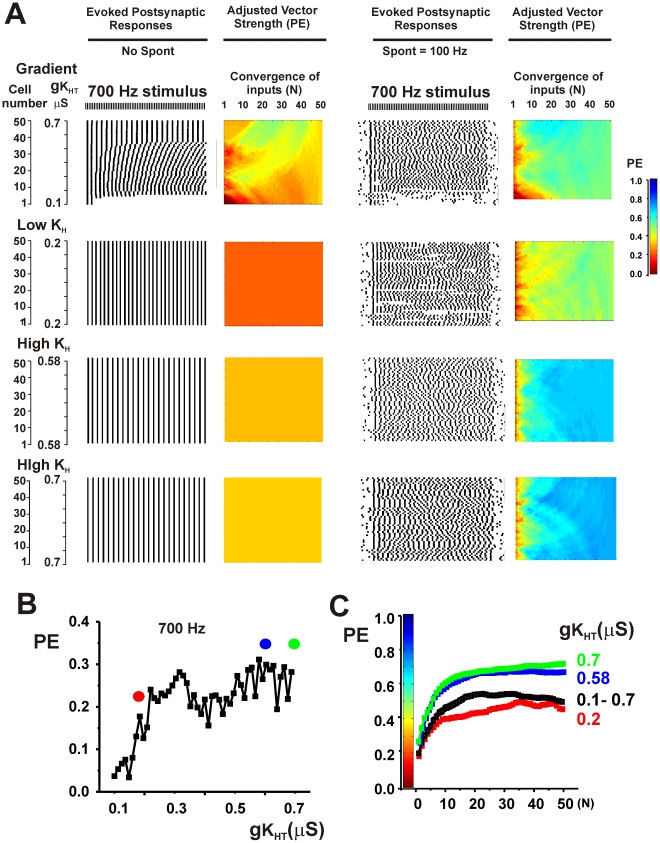
Random spontaneous activity further enhances phase vector strength PE in ensembles of neurons that express gradients of potassium currents or optimized levels of K_HT_ currents. A. Top panels show simulations for a 700 Hz stimulus applied to an ensemble with a gradient of K_HT_ current (same parameters as Fig. 4, bottom panel) with or without random spontaneous activity at a mean frequency of 100 Hz. Descriptions of panels are as for Fig. 4. The bottom three sets of panels show the effect of adjusting the value of gK_HT_ in all neurons to a low value (0.2 µS) or to high values (0.58 or 0.7 µS) in the presence or absence of spontaneous activity. B. Plot of PE against gK_HT_ for individual neurons stimulated at 700 Hz in the presence of random spontaneous activity. Dots indicate the values of gK_HT_ that were used in the lower three panels of A. C. Plots of the mean value of PE (± SEM) in the presence of spontaneous activity as a function of the number of converging inputs (N) for each of the ensembles shown in A.

**Figure 8:**
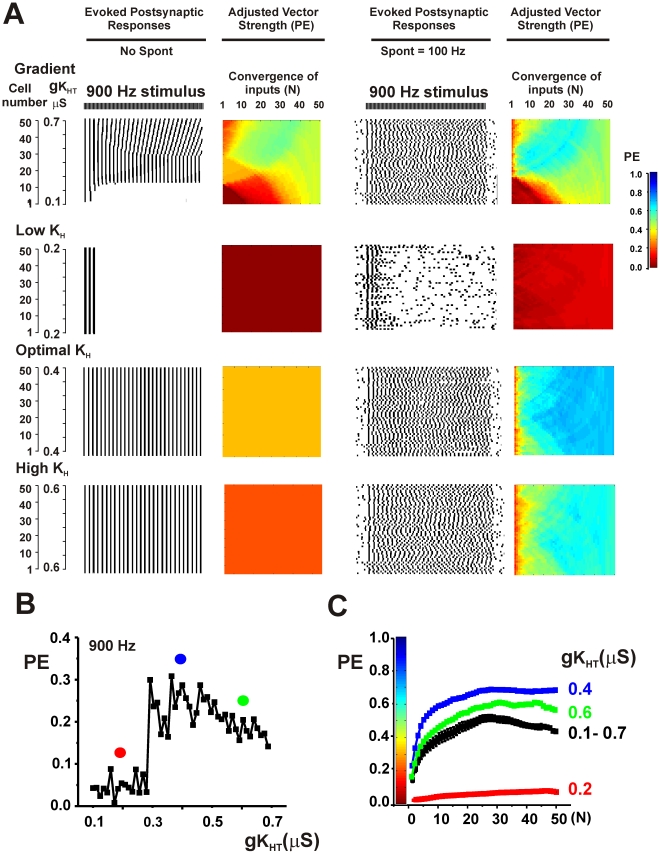
Optimization of vector strength PE can occur at intermediate values of gK_HT_. A. Top panels show simulations for a 900 Hz stimulus applied to an ensemble with a gradient of K_HT_ current (same parameters as Fig. 4, bottom panel) with or without random spontaneous activity at a mean frequency of 100 Hz. Descriptions of panels are as for Fig. 4. The bottom three sets of panels show the effect of adjusting the value of gK_HT_ in all neurons to a low value (0.2 µS), to an intermediate value (0.4 µS) or to high values (0.6 µS) in the presence or absence of spontaneous activity. B. Plot of PE against gK_HT_ for individual neurons stimulated at 900 Hz in the presence of random spontaneous activity. Dots indicate the values of gK_HT_ that were used in the lower three panels of A. C. Plots of the mean value of PE (± SEM) in the presence of spontaneous activity as a function of the number of converging inputs (N) for each of the ensembles shown in A.

Both spontaneous activity and the existence of gradients in potassium channels produce diversity in the firing patterns of individual neurons. To determine if these mechanisms occlude each other, tests were made of the effects of introducing random spontaneous activity into ensembles with gradients. In all cases, the phase vector strength of the combined synaptic outputs was improved by combining spontaneous activity with the gradient over that with either spontaneous activity or a gradient alone. This is illustrated in the top panels of Figs. 7A and 8A in which spontaneous activity (100 Hz) was added to an ensemble with the gradient first shown in the bottom panel of Fig. 4 and the ensemble was stimulated at 700 Hz or 900 Hz respectively.

In the presence of spontaneous activity, values of PE could be very substantially improved by further adjustments of high-threshold K^+^ current. Paradoxically, this occurs by “flattening” out the original gradient. Fig. 2 showed that, for any specific stimulus rate in the absence of spontaneous activity, the temporal accuracy of the output of single neurons depends on their level of K_HT_. The same is true for stimulation in the presence of spontaneous activity. For stimulation at 700 Hz, the greatest values of PE in the output of single neurons were achieved with high values of K_HT_ (Fig. 7B). The overall fidelity of the output could then be substantially improved by adjusting levels of K_HT_ in all neurons in the ensemble to those found in the neurons with the best individual responses. This is shown on the right side of Fig. 7A. The lower two sets of panels show that the accuracy of the output of the ensemble is increased by fixing K_HT_ in all the neurons at 0.58 or 0.7 µS, values at the high end of the gradient. Note that traces of the postsynaptic output for K_HT_ = 0.58 µS were shown in Fig. 5D. Conversely fixing K_HT_ in all neurons at a low value (0.2 µS) reduces temporal accuracy relative to the ensemble with full gradient. This is also shown in Fig. 7C, which plots the value of PE as a function of the number of converging inputs (N) for each of the ensembles.

Simulations were carried out for a wide variety of parameters and stimulus rates. As expected from Fig. 2, in the presence of spontaneous activity, values of PE were enhanced by adjusting levels of K_HT_ in all neurons to low values for low rates of stimulation. Conversely, for high rates of stimulation, PE was increased over that in a full gradient by adjusting K_HT_ to a high level in all neurons. Moreover, for all stimuli at high rates, the accuracy of the output of the ensemble was greater than that of individual neurons. At stimulus rates at which individual neurons responded optimally with intermediate values of K_HT_, the output of the ensemble was enhanced by adjusting K_HT_ to this intermediate value in all neurons. This is illustrated in Fig. 8 for stimulation of the same ensemble at 900 Hz. In either the presence or absence of spontaneous activity, the value of PE in individual neurons with values of K_HT_ at 0.4 µS is greater than in neighboring neurons with lower or higher K_HT_ (Figs. 2, 8B). At low levels of K_HT_ (0.2 µS), neurons are incapable of firing more than a few action potentials at the onset of the train, while higher values produce errors of timing (Fig. 8A). Adjustment of K_HT_ in all neurons to the intermediate conductance of 0.4 µS produces optimal values of PE in the output of the ensemble.

**Figure 9:**
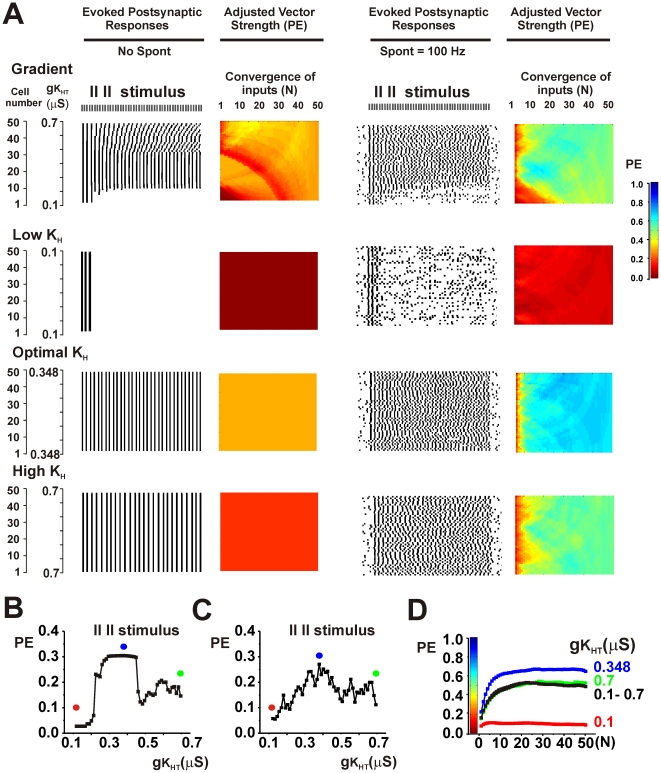
Optimization of phase vector strength PE by changes in gK_HT_ can occur for stimulus rates with multiple frequency components. A. Top panels show simulations for a stimulus comprising a two-pulse 1400 Hz burst applied repeatedly at 466.6 Hz. This was applied to an ensemble with a gradient of K_HT_ current (same parameters as Fig. 4, bottom panel) with or without random spontaneous activity at a mean frequency of 100 Hz. Descriptions of panels are as for Fig. 4. The bottom three sets of panels show the effect of adjusting the value of gK_HT_ in all neurons to a low value (0.1 µS), to an intermediate (optimal) value (0.348 µS) or to a high value (0.7 µS) in the presence or absence of spontaneous activity. B and C. Plots of PE against gK_HT_ for individual neurons stimulated with the 1400 Hz burst applied repeatedly at 466.6 Hz in the absence (B) or presence (C) of the random spontaneous activity. Dots indicate the values of gK_HT_ that were used in the lower three panels of A. C. Plots of the mean value of PE (± SEM) in the presence of spontaneous activity as a function of the number of converging inputs (N) for each of the ensembles shown in A.

In the simulations described above, the input stimuli were applied at a single rate between 100 and 1500 Hz. Auditory stimuli, however, may generally contain multiple frequency components in the phase-locking range. These components may represent either the sound frequency itself or modulation of higher frequency sounds at rates that permit locking to the envelope of the stimulus. A variety of stimulus patterns containing multiple rates were therefore then tested. Fig. 9A shows the results of stimulating the ensemble with a repeated pattern that consists of two stimuli with a 1400 Hz inter-stimulus interval followed by a pause with in inter-stimulus interval corresponding to 700 Hz. This amounts to a two-stimulus 1400 Hz burst applied repeatedly at 466.6 Hz. Even with multiple low frequency components in the stimulus train, there exist clear differences in the ability of individual neurons in a gradient to lock to the stimulus train, either in the presence or absence of spontaneous activity (Fig. 9B, C). Again, in the presence of spontaneous activity, adjustment of K_HT_ in all the neurons to the optimal value found among the individual neurons (corresponding in this case to an intermediate value of 0.348 µS) substantially enhances PE for the output of the ensemble (Fig. 9D).

## Discussion

The microsecond time scale and precision with which the auditory system operates implies that even simple aspects of an auditory stimulus, such as its temporal envelope or its location in space cannot be encoded in the activity of single neurons but must be distributed across an ensemble of neurons. The simulations presented here indicate that the accuracy that is required for estimates of timing of incoming stimuli is improved either by an orderly gradation of intrinsic excitability within the ensemble, or by random spontaneous activity. Maximal accuracy of the ensemble occurs, however, in the presence of spontaneous activity when K^+^ conductance within an ensemble of neurons can be adjusted to that present in individual neurons that initially respond with highest degree of phase-locking.

Random spontaneous activity plays two distinct roles in improving the temporal accuracy of an ensemble. The first is to increase the entrainment of the output of the entire ensemble. Consider, for example, an ensemble that is stimulated at 600 Hz but in which the individual neurons respond at 300 Hz with perfect phase-locking. In the absence of spontaneous activity, the value of Phase/Entrainment parameter PE will be 0.5, and the output of the ensemble will be a perfectly timed 300 Hz train of postsynaptic potentials. If, as was shown in Fig. 5D, spontaneous activity randomizes the timing of the onset of firing in individual neurons, the combined postsynaptic output will be a 600 Hz train of postsynaptic potentials and the PE value will be closer to 1.0. The second effect of spontaneous activity is to randomize the timing of “errors” such as those shown in Fig. 1B for high K_HT_ levels, thereby decreasing their impact on the output of the ensemble. For spontaneous activity to be effective, however, it must be random across the ensemble, with little or no correlation between individual units. A significant degree of correlation of the spontaneous activity among individual neurons is likely to be interpreted as a sensory signal, and could potentially contribute to the condition of tinnitus in humans. The high rates of spontaneous activity in the auditory nerve and in auditory brainstem neurons in the absence of sounds are known to be generated by spontaneous transmitter release from inner hair cells [16], [17], [18], [19]. For this mechanism to be effective would therefore require a relatively low degree of convergence of inputs from individual hair cells to individual brainstem neurons that constitute the presynaptic neurons in these simulations.

The contributions of a gradient of K_HT_, and the effect of modulation of levels of this current to timing accuracy are distinct from those of spontaneous activity. The level of K_HT_ in individual neurons determines both the number of errors of timing that result from the relative refractory period that follow each action potential (e.g. Fig. 1B *left*) and whether or not the neurons are able to respond to stimulation at different rates (Fig. 1B *right*). For a given stimulus pattern applied to an ensemble with a K_HT_ gradient, some neurons will respond with greater temporal accuracy than others. In addition, the diversity of intrinsic excitability in a gradient disperses the timing of the onset of firing in individual neurons, aiding the entrainment of the output of the full ensemble. In general, therefore, even in the absence of spontaneous activity the output of an ensemble with a K_HT_ gradient will be improved over that of one with a randomly-selected uniform level of K_HT_.

A central conclusion of this work is, however, that rapid adjustment of the K^+^ conductance in those neurons with a suboptimal K_HT_ to that of the optimal value for a specific stimulus pattern will, in the presence of spontaneous activity, provide the most accurate temporal representation of the input. This can result in some “flattening” of the gradient in the face of a fixed maintained auditory stimulus, as has been observed experimentally (see Fig. 4 of reference [29]). Of course a subsequent change in the stimulus pattern may require a further change in the value of K_HT_.

In this study only gradients of the high threshold Kv3.1-like K_HT_ potassium current were considered. There is an abundant experimental evidence for the existence of gradients in this channel [26], [31], [43] and auditory stimulation has been demonstrated to produce very rapid changes in Kv3.1 current levels [34], [44]. In addition, longer-term changes in auditory stimulation produce long-term changes in levels of Kv3.1 protein and K_HT_ currents in individual neurons and adjust the overall tonotopic gradient of channel expression [27], [29], [30], [31], [38], [45].

Gradients of expression along the tonotopic medial-to-lateral axis of auditory brainstem nuclei have also been described for a variety of other ion channels in many species, and are not restricted to animals with specific ranges of hearing frequency [9], [26], [28], [31], [34], [46]. In the MNTB of mammals, these include the low-threshold Kv1.1-like, Kv1.3 and K_Na_ currents [26], [32], [33]. Gradients also exist in the lateral superior olive, a target of MNTB axons [47]. Similar gradients have been characterized using both electrophysiological and molecular approaches in two different auditory nuclei of chickens and barn owls [46], [48]. Such tonotopic gradients are not confined to ion channels but have also been described for neurotransmitter receptors, synaptic proteins and signaling molecules [23], [49], [50], [51]. Gradients and adjustments in other currents, such as low-threshold K^+^ current, can also have a similar effect on accuracy of timing as changes in K_HT_ (data not shown). Thus it is likely that changes in the gradient of other parameters that influence transmission through brainstem nuclei, such as the differences in size of somata, dendrites or axons or in neurotransmitter receptors would also contribute in much the same way to enhanced processing.

The finding of gradients of expression of ion channels in the central auditory system was preceded by findings of similar gradients in sensory hair cells in the cochlea [52], [53], [54], [55], [56]. In some lower vertebrates, these differences in levels of ion channels in different hair cells serve to tune the electrical responses of the cells to specific sound frequencies [57], [58], [59], [60], [61], [62]. As in the central nervous system, gradients in the periphery are not confined to ion channels but can be found for other signaling molecules such as calcium-binding proteins and are manifest in subtle differences in the structural properties of synapses along the tonotopic axis [54], [63], [64].

To measure the accuracy of timing of the outputs of ensemble of neurons, the present simulations calculated the summed outputs as combined postsynaptic currents, as would be recorded experimentally in the voltage-clamp mode. This allowed timing information in the output to be evaluated in a way that is uncontaminated by intrinsic conductances in the postsynaptic cell. How this information is then used and shaped by intrinsic conductances in the postsynaptic cells then clearly depends on biological role of the specific circuit. Phase-locking neurons participate in circuits that encode both the frequency of incoming sound stimuli and their amplitude. Extraction of timing information would require the postsynaptic neurons to respond with high temporal fidelity to individual peaks in the postsynaptic potentials, as occurs in the AVCN and MNTB. Determination of amplitude, as occurs in the LSO, is likely to optimal when each input pulse in a train generates a postsynaptic current of equal size. Just as for timing information, this corresponds to the highest possible value for the PE parameter in the summed postsynaptic output. Thus the simulations are likely to be equally relevant to processing of timing and amplitude information.

The present findings raise the question of which specific cellular and system-level mechanisms lead to the adjustment in levels of K_HT_ in response to different patterns of stimulation. Normal physiological changes in the auditory environment produce rapid (seconds to minutes) changes in the state of phosphorylation of a serine residue in the cytoplasmic C-terminus of the Kv3.1 channel protein [34]. Specifically, the channel is normally phosphorylated in quiet environments but becomes dephosphorylated in louder environments. Such dephosphorylation results in an increase in Kv3.1 current, allowing neurons to fire at higher rates [34], [36]. Interestingly, such phosphorylation is also organized tonotopically such that, in resting neurons, a greater proportion of Kv3.1 channel is phosphorylated at the medial low-frequency aspect of the MNTB [34]. Changes in phosphorylation state appear to occur coherently across large parts of the MNTB [34], and may be mediated through cell-cell communication by messengers such as nitric oxide [44]. Thus acute changes in phosphorylation may be a key mechanism that adjusts K_HT_ currents to optimize temporal accuracy.

Longer-lasting changes in the auditory environment produce changes in the levels of Kv3.1 protein in neurons, most likely by increasing the rate of synthesis of new channel subunits [29], [30], [45]. The subset of neurons in which synthesis is enhanced depends on the frequency of the auditory stimulus that is used, and this has been shown to produce a clear change in the overall tonotopic distribution of Kv3.1 [30]. These findings suggest that, by altering the fine structure of tonotopic gradients [65], the central auditory system may be able to adjust the timing and accuracy of processing in brainstem nuclei. Perhaps in concert with feedback from higher centers, this may allow fine discriminations of patterns of inputs in a given auditory environment [66].

## Methods

### Firing patterns of individual neurons in an ensemble

Simulations of individual presynaptic neurons in an ensemble of 50 neurons were carried out using a model similar to that used previously to describe the firing patterns of MNTB neurons [34], [36], [40], [41], [67]. Responses were simulated by integration of the equation:
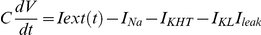
where *I_Na_* represents Na^+^ current, *I_KHT_* and *I_KL_* represent components of voltage-dependent K^+^ currents. *I_Leak_* is the leak current. Individual neurons in an ensemble were stimulated by applying step currents *I_ext(t)_* (0.2 msec, 1.5 nA) at rates of 100–1500 Hz, in the presence or absence of randomly timed stimuli with the same parameters. The capacitance C of each model neuron was 0.01 nF. Equations for *I_Na_*, *I_KHT_*, *I_KL_* and *I_L_* were identical to those in Macica et al. (2003), and are based on fits to currents in MNTB neurons. Specifically,
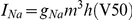


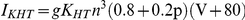


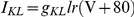


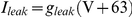



The evolution of the variables m, h, n, l and r were given by equations of the form
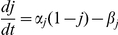
where
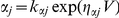


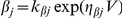
and j = m, h, n, l, r.

Kinetic parameters for the evolution of the variables m and h were g_Na_ = 0·5 µS, k_αm_ = 76.4 ms^−1^, η_αm_ = 0.037 mV^−1^, k_βm_ = 6.93 ms^−1^, η_βm_ = −0.043 mV^−1^, and k_αh_ = 0.000135 ms^−1^, η_αh_ = −0.1216 mV^−1^, k_βh_ = 2.0 ms^−1^ and η_βh_ = 0.0384 mV^−1^. For the K_HT_ (Kv3.1) current, values of *gK_HT_* were varied as described in the text, with k_αn_ = 0.2719 msec^−1^, η_αn_ = 0.04 mV^−1^, k_βn_ = 0.1974 msec^−1^ η_βn_ = 0 mV^−1^, k_αp_ = 0.00713 ms^−1^, η_αp_ = −0.1942 mV^−1^, k_βp_ = 0.0935 ms^−1^ and η_βp_ = 0.0058 mV^−1^. For the fixed “low threshold” K_L_ potassium current, *g_KL_* = 0.02 µS, k_αl_ = 1.2 ms^−1^, η_αl_ = 0.03512 mV^−1^, k_βl_ = 0.2248 ms^−1^, η_βl_ = −0.0319 mV^−1^, k_αr_ = 0.0438 ms^−1^, η_αr_ = −0.0053 mV^−1^, k_βr_ = 0.0562 ms^−1^ and η_βr_ = −0.0047 mV^−1^.

### Integration of output of ensembles of neurons

The equations for *N_tot_* (usually 50) individual neurons in an ensemble were integrated numerically for a period of 120 ms, and stimulation with external current pulses *Iext (t)* was carried out for 100 msec beginning 10 ms after the onset of integration. For each integrated trace, the times of the occurrence of the peaks of the action potentials, t_N,P_, were first calculated, where *N* is the index for the number of the neuron in the linear array of *N_tot_* neurons and *P* is the index for consecutive action potentials in the trace. The time of each action potential was defined as the time when the upstroke of the action potential crossed 0 mV. For each neuron, these were used to generate a set of step functions *F_1,N_*, *F_2,N_…F_X(N),N_*, corresponding to the times of the peaks of action potentials, where *X(N)* is the total number of action potentials evoked in neuron *N*. Thus
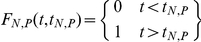
These were in turn used to generate a normalized postsynaptic current trace *S(N_start_, N_end_, t)* for the combined output of a group of neurons, where *N_start_* and *N_end_* denote the positions of the starting and ending neurons in the linear array for which the combined postsynaptic trace was calculated:
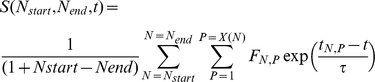



For the simulations *τ* was set at 2.0 ms.

### Calculation of PE

To calculate phase vector strength, the times of occurrences of peaks in each postsynaptic current trace *S(N_start_, N_end_, t)* were first determined. These were used to calculate a set of delays Δ(*N_start_*, *N_end_*, *u*) from the onset of each stimulus applied to the ensemble to the time of the largest peak detected in the postsynaptic trace during the subsequent inter-stimulus interval, where *u* is the index of each consecutive stimulus applied to the ensemble during the train. An initial phase vector strength V' was next calculated for the output of each linear subset of the ensemble:
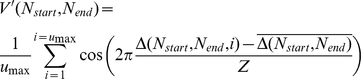
where Z is the inter-stimulus interval, *u_max_* is the number of stimuli that evoked a postsynaptic peak during inter-stimulus interval and, 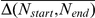 is the mean delay for these peaks.

The amplitudes of the postsynaptic current peaks *A(N_start_, N_end_, u)* for each of the stimuli in the train, as well as the maximal *S_max_(N_start_, N_end_)* and minimum *S_min_(N_start_, N_end_)* values of current were then calculated. Using the number of total stimuli that were applied to the ensemble during the train *U_tot_*, an adjusted phase vector *PE* that reflects the proportion of stimuli that evoked a postsynaptic response was then calculated. Thus
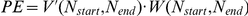
where